# Possibilities of monitoring intraocular pressure in children using EASYTON transpalpebral tonometer

**DOI:** 10.1007/s10792-021-02158-5

**Published:** 2022-01-28

**Authors:** Elena N. Iomdina, Nina Yu. Kushnarevich

**Affiliations:** Department of Refractive Pathology, Binocular Vision and Ophthalmoergonomics, Helmholtz National Medical Research Center of Eye Diseases, 14/19 Sadovaya-Chernogryazskaya St, Moscow, Russia 105062

**Keywords:** Intraocular pressure, Children, Transpalpebral scleral tonometry, Noncontact pneumotonometry, Discomfort level score

## Abstract

**Purpose:**

To compare the effectiveness of transpalpebral scleral tonometry (TPST) and corneal pneumotonometry in children, and assess the discomfort level when measuring intraocular pressure (IOP) by these methods.

**Methods:**

TPST using EASYTON tonometer (Russia) and pneumotonometry using Reichert 7 Non-contact AutoTonometer (USA) have been sequentially performed on 84 eyes (42 children aged 5–14, ave. 9.3 ± 2.7), including 64 myopic eyes (-0.5 to 6.75D), 18 hyperopic eyes (+ 0.75 to + 3.75D), and 2 emmetropic eyes. We assessed tolerance to the procedure on a five-point scale using a questionnaire which listed several criteria: discomfort, presence of pain, fear or anxiety during the procedure, the child's resistance to measurement.

**Results:**

EASYTON tonometry demonstrated repeatability of IOP indicators when measuring the same eye three times sequentially and almost the same IOP level in paired eyes of isometropic children. Pneumotonometry reveals a greater individual data variability and a more pronounced asymmetry of the paired eyes’ indicators. IOP measured using the TPST was 18.3 ± 2.3 mmHg across the whole group, 18.2 ± 2.3 mmHg in myopic, and 18.5 ± 2.3 mmHg in hyperopic children. With pneumotonometry, the corresponding indicators were 17.1 ± 3.9 mmHg, 16.9 ± 3.8 mmHg, and 18.2 ± 4.0 mmHg. The average score for the TPST (4.64 ± 0.60 points) was significantly higher than that for pneumotonometry (3.85 ± 0.90 points) (*p* < 0.05).

**Conclusions:**

TPST provides broader possibilities for IOP control in pediatric practice, yielding more reliable and accurate results than pneumotonometry, eliminating the influence of corneal thickness and irregularity on the measurement result, and ensuring a calmer behavior and more comfort of children during the procedure.

## Introduction

Registering intraocular pressure (IOP) in adults and children is an essential element of ophthalmological diagnosis and monitoring of eye diseases. While there is a large variety of ocular tonometry methods for adult patients, the range of methods for measuring IOP in children is significantly limited [1, 2]. This is accounted for by the fact that instrumental measurement of IOP is a complicated and sometimes even impracticable procedure due to the children’s negative attitude, fear, or even refusal to be examined, as well as the fact that the child's restlessness during the measurement (in particular, blepharospasm, tension of extraocular muscles, etc.) leads to unreliability of the data obtained [3–5].

In addition, the results of corneal tonometry in children depend on the corneal thickness, which changes significantly with age [6–9]. The thickness of the cornea in children between 1 and 11 years increases with age, approaching the values characteristic of adult eyes no earlier than at 9–11 years [10]. According to P. Tonnu et al. [10], the central thickness of the cornea of the child's eye has a significant influence on the results of IOP measurements carried out using Tono-PenXL and Goldman tonometers and especially using non-contact pneumotonometry. At the same time, ophthalmologists and optometrists prefer to use non-contact corneal pneumotonometry for outpatient IOP screening in children as it is the most accessible and feasible procedure under dispensary observation.

In a number of clinical situations, frequent and constant monitoring of IOP in children is required, such as congenital glaucoma or suspicion of it [11], high degree of myopia, or prolonged instillations of atropine solution to stop its progression [12, 13], prolonged instillations of steroids [14] or systemic treatment with certain medications [15], etc.

However, even the most commonly used non-contact method of corneal IOP measurement in pediatric practice, pneumotonometry, cannot always ensure the child's calm behavior during the examination and exclude his/her reaction to the measurement process (reflex blepharospasm, extraocular muscle tension, blinking, etc.), which can lead to deviation of the obtained values from the real IOP level, i.e. contribute to an increase in the measurement error [3].

Besides, using pneumotonometry in the current epidemiological situation is associated with the risk of spreading the infection [16]. Recent publications convincingly indicate an increased risk of spreading the viral disease when performing non-contact pneumotonometry: particles of tears that contain the virus easily enter the environment in the form of aerosol bubbles. This effect is cumulative and increases with higher IOP values and/or in the case of instillations of any eye drops shortly before the examination [17]. When performing the non-contact tonometry, the tear film breaks, and tear particles are released under the influence of a powerful stream of air. These aerosol bubbles can remain in the air for a long time and gradually settle on surrounding objects, including medical equipment [18].

Transpalpebral scleral tonometry (TPST) provides a real option for eliminating the drawbacks of existing methods for determining IOP in pediatric practice, including pneumotonometry since the measurement carried out transsclerally through the eyelid excludes any effect on the cornea, as well as the impact of its thickness and irregularity on the result obtained. It can be expected that the transpalpebral tonometry will provide a calmer behavior of the child during IOP measurement compared to standard pneumotonometry, which will also contribute to greater reliability and accuracy of the data.

In recent years, a new type of transpalpebral tonometer has been developed (EASYTON intraocular pressure tonometer, YIME JSC, Russia), based on the measurement of eye membranes rigidity reflecting the IOP level by determining the frequency of forced mechanical vibrations of the eyeball as an elastic system loaded with a certain mass (rod weight) under the tonometer vibrator action [19]. Transpalpebral tonometry has a specific feature as it has to alleviate the damping effect of the eyelid. The advantage of Easyton over other types of transpalpebral tonometers working on a different physical principle (rebound tonometry) is the fact that this effect is neutralized by pre-compression of the eyelid – in exactly the same way as achieved by palpation when the eyelid is pre-compressed (squeezed) with the fingers. During the measurement, the rod is placed on the eyelid in the sclera region corresponding to *corona ciliaris* in the 12-h meridian and compresses the eyelid with its weight (10 g). Thus, an integrated biomechanical “rod-eye” system is formed, the formed vibration frequency of which is determined by the actual IOP.

Contraindications to EASYTON tonometry are pathological conditions of the upper eyelid (inflammatory diseases, scars, eyelid deformity) and severe scleral pathology in the measurement area. The tonometer is calibrated in the true IOP measurement mode (Goldman scale).

**The purpose** of this work was a comparative study of effectiveness of use of transpalpebral scleral tonometry with EASYTON tonometer and corneal pneumotonometry in children, as well as a point assessment of the child's discomfort level when measuring IOP by these methods.

## Material and methods

This study was performed according to the tenets of the Declaration of Helsinki and was approved by the local ethics committee. The measurements were carried out after informed voluntary consents of the parents or legal representative of the child for it had been received.

When selecting a group of children for TPST, as well as corneal pneumotonometry, we were guided by the following criteria. Children with pathological conditions of the upper eyelid (inflammatory diseases, scars, eyelid deformity), with pronounced scleral pathology in the projection of the measurement area, with erosion, ulcers, corneal edema, those who underwent keratoplasty or had penetrating eye trauma, as well as the children with high anisometropia (more than 2 diopters) have been excluded from the examination.

Taking into account the above criteria, 42 children (84 eyes), including 23 boys and 19 girls, aged 5 to 14 years, with average age M ± SD (± m) 9.3 ± 2.7 (± 0.4) years old, have been selected for the examination, including:

18 eyes (9 children) aged 5 to 11 (average age 6.6 ± 1.9 (± 0.7)) with hyperopic cycloplegic refraction + 0.75 to + 3.75 diopters (on average for the sphere equivalent + 1.5 ± 0.9 (± 0.2) diopters).

64 eyes (33 children) aged 5 to 14 (average age 9.6 ± 1.9 (± 0.5) years) with myopic cycloplegic refraction of − 0.5 to − 6.75 diopters (on average for the sphere equivalent − 3.1 ± 0.9 (± 0.2) diopters).

2 eyes (2 children aged 5 and 8) with emmetropic cycloplegic refraction.

In addition, 6 children aged 4 to 9 (average age 7.0 ± 1.8 (± 0.7) years) with cycloplegic refraction of + 1.25 to − 3.0 diopters were examined, who were not included in the comparative statistical analysis, since it was not possible to determine their IOP by pneumotonometry (as they refused the measurement procedure).

For transpalpebral tonometry, we used an EASYTON tonometer (JSC Yelatma Instrument Making Enterprise, Russia, Registration Certificate for a medical device No. RZN 2015/2997 dated November 17, 2016).

Non-contact pneumotonometry (pneumotonometer Reichert 7 AutoTonometer, USA) has been used as a comparison method.

All children underwent a standard ophthalmological examination, which included determination of visual acuity, autorefractometry in conditions of cycloplegia (after instillations of Sol. Cyclopentolate 1%), and a thorough examination of the fundus.

**Fig. 1:**
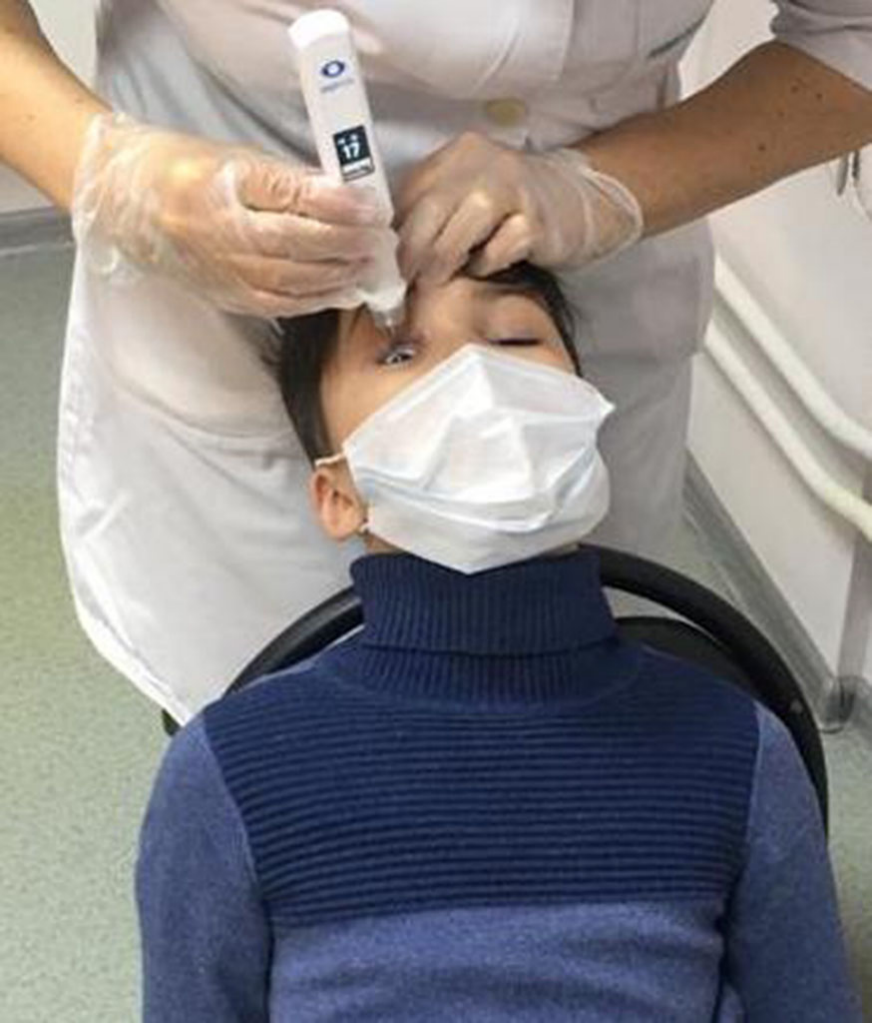
Measurement of the IOP in a child using an EASYTON transpalpebral tonometer

The IOP was measured in the sitting position, on the right and left eyes, sequentially with an EASYTON transpalpebral tonometer (see Fig. 1) and a Reichert 7 AutoTonometer pneumotonometer without use of any anesthetics.

To measure the IOP using an EASYTON tonometer, the child tilted his/her head back and fixed his/her gaze on a bright object (toy) at an angle of 45–60° to the horizontal axis; the tonometer rod was located on the upper eyelid in the scleral area corresponding to the *corona ciliaris* in the 12 o'clock meridian.

Each measurement (with an EASYTON tonometer and a pneumotonometer on the right and left eyes) was performed three times. For further analysis, the average value of these three IOP measurements was calculated for each eye with each tonometer.

After the IOP measurement procedure with an EASYTON tonometer and a Reichert 7 AutoTonometer pneumotonometer completed, the child or his/her legal representative was asked to fill out a short questionnaire, which allowed us to evaluate the measurement procedure according to the following criteria:

1. Sense of discomfort during the procedure
2. Pain during the procedure
3. Fear/anxiety during the procedure
4. The child's resistance during the procedure (this item was filled in by the doctor who performed the measurement).

The assessment was carried out on a five-point scale, where 1 point corresponded to the maximum intensity of sensation, and 5 points—to its absence.

Statistical processing of the obtained data included determination of the mean value, the standard deviation (M ± SD) and standard square error (m), as well as the Student's T-criterion. The parameter values were considered different if *p* < 0.05.

## Results

Table 1 includes the average IOP values obtained in the whole group (42 children, 84 eyes), as well as for the right (OD) and left (OS) eyes individually, using an EASYTON and a Reichert 7 AutoTonometer.

**Table 1:** The IOP values (mm Hg) for the whole group (42 children, 84 eyes), as well as for the right (OD) and left (OS) eyes individually, obtained using an EASYTON and a Reichert 7 AutoTonometer

EASYTON 18.3 ± 2.3 (± 0.3)^*^		Reichert 7 AutoTonometer 17.1 ± 3.9 (± 0.4)	
OD	OS	OD	OS
18.1 ± 2.2 (± 0.3)^†^	18.2 ± 2.3 (± 0.3)	17.0 ± 3.9 (± 0.6)^††^	17.2 ± 3.8 (± 0.6)

^*^Difference with the corresponding indicator of pneumotonometry is significant, *p* = 0.02

^†^Difference with the corresponding indicator of the fellow eyes is not significant, *p* = 0.83

^††^Difference with the corresponding indicator of the fellow eyes is not significant, *p* = 0.81

A comparative analysis of the obtained data demonstrated that the IOP values measured for the same eyes of the same children using an EASYTON tonometer turned out to be slightly higher than with pneumotonometry (*p* = 0.02). It should be noted that the results of three consecutive IOP measurements with an EASYTON transpalpebral tonometer either were exactly the same or differed by no more than 2 mm Hg, while pneumotonometry was characterized by a greater individual variety of data. In a significant part of cases (for 50 eyes, 59.5%), one or two of three pneumotonometry indicators were displayed on the tonometer display either with a * symbol (the result is doubtful) or with a "}" symbol (the result is erroneous), which indicates incorrectness or insufficient accuracy of a part of the measurements carried out.

At the same time, the survey revealed a significant difference in subjective feelings of children during the measurement and in the ease/difficulty of the procedure for the doctor, depending on the degree to which the child opposed it. The mean score for TPST on a five-point scale was 4.64 ± 0.60 (± 0.09) points, which is significantly higher than for pneumotonometry—3.85 ± 0.90 (± 0.1) points (*p* = 0.0001). It should be added that the maximum average score of 5 points for TPST (no feeling of discomfort at all) was obtained in 24 out of 42 cases (57%), while pneumotonometry obtained the maximum score only in 4 cases (9.5%).

A comparative analysis of tonometry data obtained in groups of children with myopia (Table 2) and hyperopia (Table 3) confirmed the above results and revealed some IOP features characteristic of children with these refractive errors.

**Table 2:** IOP values (mm Hg) in myopic children (33 children, 64 eyes) across the whole myopic group, as well as for the right (OD) and left (OS) eyes individually, obtained using an EASYTON and a Reichert 7 AutoTonometer

EASYTON 18.2 ± 2.3 (± 0.3)^*^		Reichert 7 AutoTonometer 16.9 ± 3.8 (± 0.5)	
OD	OS	OD	OS
18.1 ± 2.3 (± 0.4)^†^	18.3 ± 2.2 (± 0.3)	16.7 ± 3.9 (± 0.7) ^††^	17.1 ± 3.8 (± 0.7)

^*^Difference with the corresponding indicator of pneumotonometry is significant, *p* = 0.04

^†^Difference with the corresponding indicator of the fellow eyes is not significant, *p* = 0.69

^††^Difference with the corresponding indicator of the fellow eyes is not significant, *p* = 0.68

**Table 3:** IOP values (mm Hg) in children with hyperopia (9 children, 18 eyes) across the whole hyperopic group, as well as for the right (OD) and left (OS) eyes individually, obtained using an EASYTON and a Reichert 7 AutoTonometer

EASYTON 18.5 ± 2.3 (± 0.8)^†^		Reichert 7 AutoTonometer 18.2 ± 4.0 (± 1.0)	
OD	OS	OD	OS
18.5 ± 2.1 (± 0.7)^††^	18.6 ± 2.4 (± 0.9)	18.4 ± 4.0 (± 1.4)^†††^	18.0 ± 4.3 (± 1.5)

^†^Difference with the corresponding indicator of pneumotonometry is not significant, *p* = 0.82

^††^Difference with the corresponding indicator of the fellow eyes is not significant *p* = 0.92

^†††^Difference with the corresponding indicator of the fellow eyes is not significant *p* = 0.84

The IOP level in children with myopia measured by TPST was slightly higher across the group of 64 eyes than that measured by pneumotonometry (*p* = 0.04). At the same time, the pneumotonometry data were characterized by a significantly larger individual variation and a more pronounced but not statistically significant difference between the paired eyes. The data obtained with the EASYTON demonstrated (1) better repeatability when measuring the IOP in the same eye and (2) practically identical IOP levels in the paired eyes of the same child with isometropia (Table 2).

More reliable results of TPST are obviously explained by calm behavior of children and no feeling of discomfort during the procedure in most cases: in this case, the average score obtained by the survey was 4.7 ± 0.5 (± 0.1). At the same time, the corresponding indicator obtained for pneumotonometry turned out to be significantly lower and amounted to 4.0 ± 0.8 (± 0.1) (*p* = 0.0001).

In children with hyperopia, the differences between the results of transpalpebral and pneumotonometry across the whole group were statistically insignificant (*p* > 0.5), but when using EASYTON, the difference between paired eyes and the range of values were slightly lower (Table 3), which, similarly to myopia, indicates better repeatability and accuracy of TPST than pneumotonometry in this category of patients.

The analysis of the survey data obtained from children with hyperopia, as well as from children with myopia and in the transpalpebral tonometry group in general, demonstrated significantly higher (*p* = 0.025) mean scores—4.3 ± 0.8 (± 0.3) than when using pneumotonometry—3.3 ± 0.9 (± 0.3).

These data indicate better tolerability to the transpalpebral tonometry procedure than pneumotonometry for children with hyperopia, although the children in this group were much younger than the myopic children, 6.6 ± 1.9 (± 0.7) years vs. 9.6 ± 2.9 (± 0.5) years (*p* = 0.001).

A significantly better tolerability of the TPST procedure than the pneumotonometry procedure is also evidenced by the fact that it was impossible to determine IOP by pneumotonometry in 6 children (12.5%) aged 4 to 9 (average age 7.0 ± 1.8 (± 0.7) years) with cycloplegic refraction + 1.25 to − 3.0 diopters because they refused to undergo the measurement. This group of children was not included in the comparative statistical analysis. At the same time, none of the examined children refused transpalpebral tonometry.

## Discussion

Literature data demonstrates that the use of a lower-contact technique—point rebound tonometry (with an Icare tonometer) for measuring IOP in children (when treating myopia with atropine) instead of contact tonometry according to Goldman does not lead to a significant decrease in accuracy: in 76.1% of cases, the discrepancy between the two IOP tonometers was within 2 mm Hg [20]. At the same time, the rebound tonometry (with an Icare tonometer) was even better tolerated by children (especially under 6 years) than pneumotonometry: in the first case, the IOP was successfully measured in 88.9% of children, and in the second case—only in 72.2% [5].

At the same time, the data of rebound tonometry (Icare tonometer) obtained in children with congenital glaucoma, systematically diverged towards overestimation with the results of measuring IOP with a portable Perkins applanation tonometer [21]. The difference between the data of these tonometers was more pronounced in children with thicker corneas.

Ophthalmic tonometry performed by four different devices: an Icare tonometer, a pneumotonometer, a Maklakov applanation tonometer, and a TVGD-1 transpalpebral tonometer in healthy children and children with congenital glaucoma demonstrated that the results of measurements performed by all of the above methods depended on thickness of the cornea, with the exception of transpalpebral tonometry, which only moderately depended on the length of the anteroposterior axis of the eye [3]. A positive experience of using the TVGD-1 transpalpebral tonometer in children with myopia and in children with increased IOP was somewhat earlier presented by E.E. Tugeeva and T.N. Vorontsova [22]. The authors compared the data of the transpalpebral tonometer and the Maklakov applanation tonometer and demonstrated a statistically insignificant difference between the indicators obtained in the respective groups of the children.

The technical parameters of the EASYTON tonometer used for transpalpebral tonometry in our study were improved (as compared to TVGD-1) based on experimental research in which the tonometric IOP values were compared with the true manometric pressure inside the eye. The results of this study made it possible to improve the measurement accuracy [23].

Indeed, the repeatability of IOP indices when measured three times sequentially using an EASYTON tonometer on the same eye and almost the same IOP level in paired eyes with isometropia indicate the reliability and higher accuracy of the results obtained in comparison with pneumotonometry, which was characterized by a greater individual scatter of data and a more pronounced asymmetry of indicators in the paired eyes.

The results of the survey aimed at assessing tolerance and comfort of the measurement procedure for children demonstrated significant advantages of transscleral transpalpebral tonometry over corneal non-contact pneumotonometry: in most cases, the children did not feel discomfort and calmly accepted tonometry with the EASYTON apparatus. The mean score for TPST on a five-point scale, which was 4.64 ± 0.60 (± 0.09) points, was statistically much higher than the relevant score for pneumotonometry, which was 3.85 ± 0.90 (± 0.1) points (*p* = 0.0001). At the same time, more than half of the children (57%) did not feel any discomfort at all during transpalpebral tonometry, while during pneumotonometry, only 9.5% assessed the measurement procedure in such a way. Moreover, none of the children involved in the study rejected transpalpebral tonometry, in contrast to pneumotonometry, which failed for 6 of 48 children (12.5%).

In our opinion, the calm behavior of the child during transpalpebral tonometry is an important advantage of this technique, which contributes to obtaining more reliable data. It should also be added that in certain cases (conditions after keratosurgery, keratopathies with pronounced edemas or inflammatory manifestations), corneal tonometry is inapplicable at all. In such cases, transpalpebral tonometry may be used instead of palpatory IOP control.

Another important advantage of transpalpebral tonometry over pneumotonometry is a decreased risk of transmission of a viral infection caused by the fact that tear particles that contain a virus, in the form of aerosol bubbles formed under the influence of a pneumoimpulse, can survive in the air and on surrounding objects for a long time [18]. At the same time, the sensor of EASYTON tonometer, which works on a completely different physical principle, has no contact with the surface of the eye at all (it only contacts the upper eyelid skin) and can be easy disinfected.

Limitations of our study include the need to compare the 2 tonometers (EASYTON and pneumotonometer) in children with IOP pathology (such as glaucoma) and the need to compare the 2 tonometers with the gold standard Goldmann applanation. As concerns the first limitation, we refer to previous work by E.Tugeeva et al. [3], in which the comparison of the results of tonometry performed using the Icare tonometer, transpalpebral tonometer TVGD-01, pneumotonometry, and Maklakov tonometer in healthy children and patients with congenital glaucoma showed that tonometry results for the same healthy children and for children with congenital glaucoma, though different, are not statistically reliable (*p* > 0.05). We used an improved version of the transpalpebral tonometry (EASYTON) and assume that a similar comparative study is likely to yield a close result–although of course, this assumption has to be verified, which we plan to do in a subsequent study. The same conclusion can be made for the comparison of tonometry results in children by EASYTON and Goldman tonometry in children.

## Conclusion

A comparative study of effectiveness of the transpalpebral scleral tonometry using an EASYTON tonometer and corneal pneumotonometry in children, as well as scoring of the child's discomfort level when measuring IOP by these methods, which were carried out for the first time, revealed significant advantages of the transpalpebral scleral tonometry. In our opinion the transpalpebral scleral tonometry provides wider options for IOP control in pediatric practice, since it allows obtaining more reliable and accurate results than corneal pneumotonometry, eliminating the influence of thickness and irregularity of the cornea on the measurement result and ensuring a calmer behavior of children and their comfort during the procedure.

## Data Availability

All primary data can be presented upon request.

## References

[1] Tugeeva EE, Brzheskiy VV (2016). Features of measurement of intraocular pressure in children. Ophthalmology Journal.

[2] Bresson-Dumont H (2009). La mesure de la pression intra-oculaire chez l'enfant [Intraocular pressure measurement in children]. J Fr Ophtalmol.

[3] Tugeeva EE, Vorontsova TN, Brzheskiy VV, Zaytseva MV (2015). The influence of the fibrous capsule main parameters on the results of different methods of ophthalmotonometry in children. Ros pediatr ophthal’mol.

[4] Chan WH, Lloyd IC, Ashworth JL, May K, Bhojwani RD, Biswas S (2011). Measurement of intraocular pressure in children in the UK. Eye (Lond).

[5] Kageyama M, Hirooka K, Baba T, Shiraga F (2011). Comparison of ICare rebound tonometer with noncontact tonometer in healthy children. J Glaucoma.

[6] Muir KW, Jin J, Freedman SF (2004). Central corneal thickness and its relationship to intraocular pressure in children. Ophthalmology.

[7] Doughty MJ, Laiquzzam M, Müll A, Oblaker E, Norman FB (2002). Central corneal thickness in European (white) individuals, especially children and the elderly, and assessment of its possible importance in clinical measures of intraocular pressure. Ophthalmic Physiol Opt.

[8] Krzyżanowska-Berkowska P, Asejczyk-Widlicka M, Pierscionek B (2012). Intraocular pressure in a cohort of healthy Eastern European schoolchildren: variations in method and corneal thickness. BMC Ophthalmol.

[9] Bradfield YS, Kaminski BM, Repka MX (2012). Comparison of Tono-Pen and Goldmann applanation tonometers for measurement of intraocular pressure in healthy children. J Am Assoc Pediatr Ophthalmol Strabismus.

[10] Tonnu PA, Ho T, Newson T, El Sheikh A (2005). The influence of central corneal thickness and age on intraocular pressure measured by pneumotonometry, non-contact tonometry, the Tono-Pen XL, and Goldmann applanation tonometry. Br J Ophthalmol.

[11] Flemmons MS, Hsiao YC, Dzau J, Asrani S, Jones S, Freedman SF (2011). Icare rebound tonometry in children with known and suspected glaucoma. J AAPOS.

[12] Wu J, Tzu-En C-C, Chen H-S (2012). Does atropine use increase intraocular pressure in myopic children?. Optom Vis Sci.

[13] Lachkar Y, Bouassida W (2007). Drug-induced acute angle closure glaucoma. Curr Opin Ophthalmol.

[14] Musleh MG, Bokre D, Dahlmann-Noor AH (2020). Risk of intraocular pressure elevation after topical steroids in children and adults: a systematic review. Eur J Ophthalmol.

[15] Hadjikoutis S, Morgan JE, Wild JM, Smith PE (2005). Ocular complications of neurological therapy. Eur J Neurol.

[16] Lu CW, Liu XF, Jia ZF (2020) 2019-nCoV transmission through the ocular surface must not be ignored. The Lancet 24. Available at: https://www.sciencedirect.com/science/article/pii/S014067362030313DOI:10.1016/S0140-6763(20)30313-5 10.1016/S0140-6736(20)30313-5 PMC7133551 32035510

[17] Yan T, Li Ch, Chen Y (2020). Effect of intraocular pressure on aerosol density generated by noncontact tonometer measurement. J Glaucoma.

[18] Britt JM, Clifton BC, Barnebey HS (1991). Microaerosol formation in noncontact “air-puff” tonometry. Arch Ophthalmol.

[19] Dykin VI, Ivanishchev KV, Kornev NP, Mikheev AA, Solomakha VN (2013). Device for calibration of the TVGD-01 dynamic tonometer. Biomed Eng.

[20] Weng J, Tsai IL, Kuo LL, Tsai CY, Woung LC, Hsiao YC (2017). Intraocular pressure monitoring by rebound tonometry in children with myopia. Taiwan J Ophthalmol.

[21] Martinez-de-laCasa JM, Garcia-Feijoo J, Saenz-Frances F (2009). Comparison of rebound tonometer and Goldmann handheld applanation tonometer in congenital glaucoma. J Glaucoma.

[22] Tugeeva EE, Vorontsova TN (2013). Possibilities of transpalpebral tonometry by TVGD-1 in children. Rossijskij meditsinskij zhurnal.

[23] Iomdina EN, Klevtsov EA, Ivanishchev KV (2019). Experimental simulation for determining optimal design parameters of a transpalpebral tonometry sensor. Vestnik oftal'mologii.

